# Co-creating palliative care education across contexts: faculty experiences from a Ghana–Canada partnership for primary health care strengthening

**DOI:** 10.3389/fmed.2026.1860858

**Published:** 2026-07-21

**Authors:** Mawuli Kotope Gyakobo, Abigail Kusi Amponsah, Michael Owusu-Ansah, Anita Eseenam Agbeko, Perditer Okyere, Arti Singh, Amy Nolen, Rachel Wortzman, Adam Goldman, Sandy Shamon, Jamie Meuser, Paolo Mazzotta, Katherine Whitehead, Salman Bahammam, Jennifer Wilson, Katherine Rouleau, Kirsten Wentlandt

**Affiliations:** 1 Department of Internal Medicine and Therapeutics, School of Medical Sciences, College of Health and Allied Sciences, University of Cape Coast, Cape Coast, Ghana; 2 Department of Public Health Nursing, School of Nursing and Midwifery, College of Health Sciences, KNUST, Kumasi, Ghana; 3 Department of Community Health, School of Medical Sciences, College of Health Sciences, KNUST, Kumasi, Ghana; 4 Department of Surgery, School of Medical Sciences, College of Health Sciences, KNUST, Kumasi, Ghana; 5 Renal Unit, Department of Medicine, School of Medical Sciences, College of Health Sciences, KNUST, Kumasi, Ghana; 6 Department of Epidemiology and Biostatistics, School of Public Health, College of Health Sciences, KNUST, Kumasi, Ghana; 7 Division of Palliative Care, Department of Family and Community Medicine, University of Toronto, Toronto, ON, Canada; 8 Division of Palliative Medicine, Sunnybrook Health Sciences Centre, Toronto, ON, Canada; 9 Temmy Latner Centre for Palliative Care, Sinai Health System, Toronto, ON, Canada; 10 Palliative Medicine Unit, Department of Internal Medicine, King Saud University, Riyadh, Saudi Arabia; 11 Department of Family and Community Medicine, University of Toronto, Toronto, ON, Canada; 12 St. Michael's Hospital, Unity Health Toronto, Toronto, ON, Canada; 13 Division of Palliative Care, Department of Supportive Care, University Health Network, Toronto, ON, Canada

**Keywords:** capacity building, education, global health, interprofessional, palliative care, primary health care, program development, sustainable development goals

## Abstract

**Introduction:**

Palliative care education developed in high-income settings often requires significant contextual adaptation to address the needs of diverse clinical and cultural environments. Globally, strengthening palliative care is increasingly recognized as critical to achieving the health-related sustainable development goals (SDGs), Universal Health Coverage (UHC), and strong primary health care (PHC)-oriented systems. Despite this, access to palliative care remains limited globally, particularly in low-and middle-income countries. Co-creation provides a collaborative approach to developing relevant and equitable curricula that can support the integration of palliative care into primary care and advance progress toward UHC and the SDGs; however, little is known about how this process unfolds in transnational partnerships, particularly in West Africa. This study aims to describe how Ghanaian and Canadian faculty experienced the co-creation and delivery of a palliative care training programme.

**Materials and methods:**

We conducted a qualitative study using semi structured interviews with faculty from Kwame Nkrumah University of Science and Technology (KNUST; Ghana) and the University of Toronto (UofT; Canada) who co-developed and facilitated a palliative care course between 2023 and 2025 under the Africa Health Collaborative (AHC). Purposive sampling included all eligible faculty participants. Interviews were conducted in person, audio-recorded, transcribed, de-identified, and analyzed using inductive thematic analysis. Sampling continued until conceptual depth was reached.

**Results:**

Ten faculty described five interconnected themes to characterize their co-creation experience. Navigating culture and context required ongoing adaptation of teaching approaches and clinical content to ensure relevance. The co-creation process evolved from initial uncertainty and vulnerability toward trust, cohesion, and shared ownership. Communication was central, serving both as a pedagogical focus and as a foundation for collaboration. Participants described professional and personal growth, including expanded perspectives and enhanced teaching practices. Co-creation was also viewed as a promising model for future curriculum development, although barriers such as limited preparation time, technological challenges, and initial apprehension were identified.

**Conclusion:**

Within this Ghana–Canada partnership, co-creation functioned as a relational and adaptive process that supported contextually grounded curriculum development and collaborative learning. These findings provide insight into the experiential and relational dimensions of co-creation in global health education and may inform similar partnerships, while highlighting the importance of contextual adaptation in resource-diverse settings.

## Introduction

Palliative care education is increasingly recognized as essential to strengthening health systems globally, including advancing Universal Health Coverage (UHC) and reinforcing primary health care (PHC), especially in low- and middle-income settings where the burden of serious illness, non-communicable disease and aging populations is rising, along and where access to both high-quality, comprehensive primary care and specialized services remains limited (1). In PHC-oriented health systems, primary care is understood as the entry point and the foundation of integrated health services optimally expected to address 80% of a population's health needs across the spectrum from health promotion and prevention, to acute and chronic care, rehabilitation and palliation (1).

Despite this growing need for the integration of palliative care in primary care, educational resources and curricula are often developed in high-income settings and transferred to other contexts with minimal or no adaptation. Previous studies have shown that this approach often fails to account for differences in health system infrastructure, resource availability, cultural beliefs, and social practices surrounding serious illness and end-of-life care, thereby limiting the effectiveness and sustainability of these educational programs (2–4).

Against this backdrop, there has been a growing shift toward co-creation as another way to traditional top-down models of curriculum transfer as an alternative paradigm in global health education. This approach emphasizes shared decision-making, mutual learning, and collaborative curriculum design (5), and is increasingly being recognized as a promising strategy for developing programs that are contextually grounded and equity-oriented, with potential to support integration of palliative care into PHC-oriented systems and progress toward UHC and the sustainable development goals (SDGs). Despite growing interest there is limited evidence on how co-creation unfolds in practice within transnational partnerships and within the context of palliative care education in West Africa (6–8).

Emerging literature from global health partnerships further highlights the importance of contextual adaptation and bidirectional collaboration in educational initiatives between high-income and low- and middle-income countries. For example, work in Nigeria has demonstrated the critical role of adapting palliative care curricula to local clinical realities and health systems (9), while other international collaborations have emphasized the value of sustained partnerships in building workforce capacity and supporting specialty training (10–12). These studies underscore both the promise and complexity of transnational educational partnerships, while also highlighting ongoing gaps in understanding how collaborative processes such as co-creation unfold in practice.

Despite growing interest there is limited evidence on how co-creation unfolds in practice within transnational partnerships and within the context of palliative care education in West Africa (6–8). Existing literature has largely focused on curriculum development and implementation, with limited attention to the lived experiences of faculty engaged in these processes. In particular, little is known about how relational, cultural, and pedagogical dynamics—including communication, vulnerability, and partnership development—unfold within transnational palliative care education initiatives. Understanding these processes is critical to informing the design of equitable, contextually relevant, and sustainable global health education partnerships.

In response to the rising need for palliative care education in Ghana, faculty from Kwame Nkrumah University of Science and Technology (KNUST) in Ghana and the University of Toronto (UofT) in Canada jointly developed and facilitated a continuing education program through an ongoing institutional partnership under the Africa Health Collaborative (AHC). This collaboration provided a valuable opportunity to explore the interpersonal, cultural, and pedagogical processes that shape co-creation across diverse contexts.

The purpose of this study was to explore the experiences of Ghanaian and Canadian faculty involved in the co-creation and delivery of a palliative care education program, with particular attention to the relational, cultural, and pedagogical processes that shaped the collaboration.

To guide this study, the following research questions were developed: (1) How do Ghanaian and Canadian faculty describe their experiences of co-creating and delivering a palliative care education program? (2) What processes shape the co-creation experience?

## Methods

### Study design

We conducted a descriptive qualitative study involving faculty who developed, implemented, facilitated, and evaluated the palliative care course in Ghana. Our study design utilized a social-constructivist framework due to its emphasis on the co-construction of knowledge and reality through social interactions, cultural contexts, and shared human experiences rather than the pursuit of objective discovery (13). This approach aligns with the study's focus on co-creation, which emphasizes relational processes, shared meaning-making, and the interpretation of experiences within a specific social and institutional context. This framework informed the study design, data collection, and analysis, with the aim of understanding how faculty experienced and interpreted the process of co-creating a palliative care curriculum. This study was approved by the KNUST Committee on Human Research, Publication, and Ethics (ID: CHRPE/AP/1225/24). Participation in the study was voluntary. Potential participants were informed of the purpose of the study, and their right to decline participation or withdraw at any time without consequence. Informed consent was obtained prior to participation. No financial or material incentives were provided for participation. All interview data were treated confidentially and de-identified prior to analysis. Audio recordings and transcripts were stored securely and accessed only by members of the research team. Data were used solely for the purposes of this study.

### Co-creation of palliative care education by Ghanaian and Canadian faculty

The collaboration between KNUST and UofT is situated within the Africa Health Collaborative, a multi-institutional initiative designed to strengthen health systems through reciprocal, cross-border partnerships. Within this framework, KNUST and UofT embarked on a multi-year partnership with the shared objective of jointly developing continuing professional education programs for primary care providers in Ghana. Central to this initiative was the commitment to co-creation: a process emphasizing shared leadership, mutual learning, and the integration of diverse institutional and cultural perspectives (14). The development of a palliative care short course emerged as one of the early outputs of this collaborative effort with a goal to co-create an educational program targeted for primary healthcare workers to improve palliative care capacity in Ghana.

### Establishing the co-creation partnership

The co-creation process began with the deliberate selection of faculty from both institutions based on their disciplinary expertise, clinical experience, and familiarity with palliative care practice in their respective contexts. Faculty were drawn from the departments of palliative care, internal medicine, family medicine, nursing and public health. This interdisciplinary composition ensured that the working group reflected a broad spectrum of clinical and academic perspectives.

Following its formation, the joint faculty group functioned as a binational working team responsible for conceptualizing, designing, implementing, and evaluating the program. Co-creation was operationalized through shared decision making at every stage of program development. In contrast to hierarchical model in which one institution directed the process, the partnership emphasized co-ownership of the curriculum and pedagogical framework, ensuring an equitable and collaborative program delivery.

### Co-design processes and collaborative structures

The co-design period started approximately 6 months before the initial delivery of the module in 2023. During this period, the working group engaged in an iterative and adaptive planning process characterized by frequent communication and progressive refinement of program components. Faculty met regularly through virtual platforms, with the frequency of meetings increasing from monthly to weekly as the delivery date approached. These meetings created the conditions for dialogue, negotiation, and collaborative problem solving, enabling the team to reconcile differing institutional practices, teaching traditions, and resource considerations.

The co-creation process extended beyond content development to include the broader structure of the learning experience. A process was established to elicit complex clinical scenarios, led by faculty, but defined by the learners who contributed to case building activities and discussions in real-life. Faculty collaboratively determined the sequencing of modules, the integration of experiential learning components, and the balance between didactic and interactive teaching methods. Ghanaian faculty provided essential contextual grounding, ensuring that the curriculum aligned with local clinical realities, health system structures, and sociocultural dimensions of serious illness and end of life care. Canadian faculty contributed additional content expertise, and pedagogical planning. The resulting curriculum thus emerged as a negotiated and jointly constructed educational model rather than the importation of an externally developed course.

### Iterative adaptation and reflexive practice

A defining feature of the co-creation approach was its emphasis on continuous learning and adaptation. Following each module delivery, the joint faculty team engaged in-person debriefs in Ghana, followed by subsequent virtual meetings to review evaluation data and inform ongoing revisions. This reflective practice allowed the course to evolve responsively in real time in response to learner feedback, faculty observations, and emerging needs.

### Co-creation of evaluation processes

The evaluation strategy was also co-developed alongside the curriculum. Daily class exit surveys were developed and implemented to capture immediate learner feedback. Leaders and faculty from both institutions jointly designed the program exit survey instruments and focus group guides, ensuring that the tools captured both the pedagogical dimensions of the course and the dynamics of the co-creation process. Ghanaian data collection teams contributed contextual insight and led participant engagement activities, while both institutions collaborated on data analysis and interpretation. This collaborative approach to evaluation reinforced transparency, cultural appropriateness, and mutual accountability in assessing programme outcomes.

### Setting, population and recruitment

This study was conducted during the delivery of the course offered in 2023, 2024, and 2025. They took place in different locations/regions (Kumasi, Cape Coast and Carpenter) in Ghana. The sampling frame consisted of all faculty participating in the collaboration across both Ghanaian and Canadian institutions, and all eligible individuals were invited to participate. Eligible participants were faculty members from Ghana and Canada who had actively participated in the facilitation of at least one module of the palliative care course. Faculty were recruited in person during the course delivery periods. Participants and several members of the research team were part of the same faculty group involved in the co-creation and delivery of the program, creating a context in which researchers and participants shared professional roles within the partnership. To minimize coercion and reduce potential bias, recruitment and data collection were conducted by trained research staff on the KNUST Monitory and Evaluation Team who were not involved in the development or facilitation of the palliative care course. Eligible faculty were approached individually at the teaching venue. The study purpose was explained, and their right to decline participation or withdraw at any time without consequence. Questions were addressed, and informed verbal consent was obtained prior to participation in accordance with approved ethical procedures. Ten faculty members consented to participate, representing the majority of eligible participants.

Interviews were conducted onsite in private settings at either the hotel or the teaching venue to ensure confidentiality and convenience for participants. This approach allowed interviews to take place immediately following teaching activities, enabling faculty to reflect on their experiences in real time. All interviews were conducted face-to-face by experienced qualitative interviewers and audio recorded with participant permission.

### Data collection, measures and analysis

Data were collected through semi-structured interviews guided by an interview protocol co-developed by academic leads from both KNUST and the UofT. The guide was designed to elicit rich descriptions of faculty experiences with the co-creation process, including their perspectives on collaborative curriculum development, interdisciplinary and cross-cultural teamwork, and their experiences teaching the course. Questions also explored faculty roles during course delivery, interactions with students, perceptions of the learning environment, and experiences with logistical and administrative support at the teaching sites. The semi-structured format allowed interviewers to probe for further detail while enabling participants to reflect on aspects of co-creation that were most relevant to them. It was reviewed within the research team to ensure clarity, relevance, and cultural appropriateness prior to use. The guide was not formally pilot-tested prior to data collection. While not based on a previously validated instrument, the development of the guide was informed by the study aims and relevant literature on co-creation and global health education. Given that the study focused on a single cohort of faculty involved in the co-creation process, some of whom were also part of the broader research team, triangulation across independent data sources or participant groups was not undertaken.

Sample adequacy was determined using the principle of conceptual depth (15), whereby recruitment concluded once the research team judged that additional interviews were unlikely to yield new or conceptually distinct insights.

Interviews were conducted using a flexible, conversational approach to support participant comfort and encourage open dialogue. Rapport was facilitated through the use of open-ended questions, active listening, and allowing participants to guide the depth and direction of discussion. Interviewers were trained research staff not involved in program delivery, which helped minimize potential power differentials. Where clarification was needed, interviewers used probing, rephrasing, and follow-up questions to ensure understanding and allow participants to elaborate on their responses. Participants were given time to reflect and expand on their experiences throughout the interview. Interview transcripts were not returned to participants for comment or correction.

The individual face-to-face interviews were audio-recorded, de-identified with an assigned study identification number and transcribed verbatim prior to analysis. All interview data were treated confidentially. Audio recordings and transcripts were stored securely and accessed only by members of the research team. Transcripts were not returned to participants for comment or correction.

We employed a thematic analysis approach to examine participants' understanding and experiences of co-creation, attending to patterns related to cultural context, relational dynamics, pedagogical processes, and perceived outcomes of the partnership (16). Codes, categories and themes were inductively generated by the team to reflect the shared experiences of the faculty members. Two members of the research team (KW and RW) independently reviewed and coded transcripts, and conflicts were resolved through discussions to achieve consensus. Consistent with a social constructivist approach, analysis focused on identifying shared patterns of meaning across participants while recognizing the influence of social context and interaction on these experiences (13). Coding was conducted using an inductive approach, with initial codes generated from close reading of the transcripts. These codes were then organized into broader categories and iteratively refined into themes through ongoing discussion among the research team. Themes were reviewed and refined to ensure they accurately reflected patterns in the data and were grounded in participants' accounts. In contrast to approaches that emphasize coding reliability through statistical agreement, reflexive thematic analysis does not seek inter-rater reliability as a marker of rigor. Instead, it prioritizes reflexive engagement with the data and the generation of meaning through researcher interpretation.

Several members of the research team, including KW and RW, were also involved as faculty in the co-creation and delivery of the course under study. Consistent with a social constructivist approach (17), we recognized that these dual roles may shape data interpretation. To address this, coding was conducted independently prior to reflexive comparison, and analytic decisions were discussed within the broader research team, including members not directly involved in program delivery, to support critical reflection and minimize individual bias. Data interpretation emphasized capturing shared patterns across participants rather than privileging insider perspectives. In addition, recruitment and data collection were conducted by independent research staff not involved in course delivery, further supporting analytic rigor. While formal reflexive bracketing was not undertaken, researchers engaged in ongoing reflexive practice throughout the study, considering how their perspectives and roles may have influenced interpretation.

Given the defined and limited cohort of eligible participants, sampling was comprehensive rather than iterative, and analysis focused on achieving depth of understanding rather than formal data saturation. Reporting of this study was informed by established qualitative research reporting standards, including the Consolidated Criteria for Reporting Qualitative Research (COREQ).

## Results

A total of 10 interviews were completed; six with Ghanaian faculty and four with Canadian faculty. Seventy percent of the faculty were female; one faculty member had a background in nursing and the remainder in medicine. Detailed demographic characteristics (e.g., age, gender, years of professional experience) were not systematically collected as part of this study. Participants were all faculty members involved in the co-creation and delivery of the program across Ghanaian and Canadian institutions. Five interconnected themes were generated: (1) Navigating culture and context, (2) Co-creation as an evolving relational process, (3) Communication as a pedagogical and collaborative core, (4) Professional and personal growth, and (5) Expanding the concept and future potential of co-creation. These themes reflect the complex interpersonal, cultural, pedagogical, and structural dynamics of building a transnational educational partnership.

### Navigating culture and context

Faculty described entering the teaching environment with varying levels of cultural familiarity and confidence, particularly among Canadian faculty who expressed uncertainty about the relevance of their examples and clinical recommendations in a Ghanaian context. As one faculty reflected,

“*One challenge I sometimes experience is wondering whether what I am teaching is fully relevant.” (FAC3)*.

Others noted concerns about medication availability and the cultural applicability of clinical scenarios, describing moments when they had to,

“...*pause to consider whether certain medications are available here or whether cultural aspects make the example applicable*.”* (FAC3)*

Storytelling and case-based learning emerged as especially powerful pedagogical tools. Learners,

“...*gravitated toward storytelling… engagement increased significantly when real-life cases were discussed (FAC5).”*

and

“*...when storytelling begins, participants become very attentive (FAC7).”*

Faculty highlighted how culturally resonant examples helped illuminate the emotional, spiritual, and psychological dimensions of palliative care that are particularly salient in Ghanaian practice,

“...*examples are drawn from our local context (FAC8).”*

Developing comprehensive clinical cases with students in class allowed all faculty to use contextual real-life scenarios within their teaching. One faculty stated,

“*We created case-based scenarios with ethical dilemmas to stimulate discussion… facilitated meaningful conversations (FAC3).”*

Over time, differences in cultural and clinical norms became a strength with a Ghanaian faculty member emphasizing how exposure to international perspectives expanded their understanding of global practice, explaining,

“*There are so many things that we need to learn from other people… that should be the way forward in adding voices to someone's voice (FAC2)”*

Likewise, repeated interaction fostered relational ease, as one faculty noted,

“*We've gotten to know ourselves a lot more… the ice is broken, and we are able to coordinate better than we used to (FAC6).”*

### Co-creation as an evolving relational process

Co-creation itself was described as a transformative and evolving process marked by early uncertainty and gradual movement toward trust, shared responsibility, and mutual learning. Several faculty, especially Ghanaian faculty newer to co-creation methodologies, described their initial surprise:

“*I never knew that we could also co-create a curriculum… this was my first time as well (FAC1).”*

As relationships deepened, co-creation became increasingly fluid, characterized by frequent reciprocal feedback loops and collaborative refinement of materials. Faculty described regular meetings, iterative content development, and dynamic adjustments in response to learner needs. One faculty highlighted this iterative nature:

“*You should be ready for a lot of teamwork (FAC1).”*

Others emphasized the reciprocity underpinning successful co-creation:

“*We shared ideas and things that they didn't know… they also had to share with us (FAC2).”*

Mutual support within the teaching team was essential. Faculty noted how colleagues stepped in when others were uncomfortable with specific content:

“*If I didn't feel comfortable teaching a slide or two, they would pick it up for me. (FAC9)”*

Faculty observed that over time the group improved the way they worked together. One faculty described.

“*We are a lot more cohesive… we've gotten to know each other more than we used to (FAC6).”*

Over time, this collaboration fostered a sense of collective identity, captured by one faculty's remark:

“*We've gotten used to each other… we are becoming more like a family (FAC4).”*

Co-creation also demanded flexibility, particularly in navigating culturally sensitive content such as physician-assisted dying, which one faculty described as,

“*not yet legal in Ghana… it was quite insightful (FAC4)”*

to explore differences while maintaining contextual appropriateness.

Faculty emphasized that successful co-creation requires partnership and the need to develop relationships before teaching begins. Several advocated for more structured pre-course orientation sessions or earlier faculty contact, suggesting that

“*one of the things I would do differently is connect with the faculty here earlier (FAC9)*.”

### Communication as a core pedagogical and professional value

Communication emerged as a central theme across both the teaching process and the palliative care content itself. Faculty repeatedly emphasized that co-creation required openness, transparency, and comfort with critique, noting that effective collaboration depends on

“*good communication skills… because you are going to be doing this with many different backgrounds (FAC1)*.”

Within the course, communication was viewed not simply as a clinical skill but as a core professional competency central to palliative care practice. One faculty summarized this sentiment:

“*If I had to pick one thing… it would be communication skills. A lot of healthcare and medicine is art… the art piece is sometimes not well trained (FAC10).”*

Teaching strategies intentionally emphasized communication: roleplays, case-based discussions, ethical vignettes, and structured conversation tools. As another faculty explained,

“*The entire first day was about communication tools and case scenarios… I think they will walk away remembering these skills (FAC10).”*

Communication also played a vital role in bridging cultural differences and building mutual trust within the multinational teaching team. Faculty described how regular check-ins, collaborative troubleshooting, and honest discussion of uncertainties strengthened the partnership and enhanced co-teaching cohesion.

### Professional and personal growth

The co-creation and co-delivery experiences fostered substantial professional and personal growth for faculty from both Ghana and Canada. Canadian faculty described the opportunity as

“...*a privilege… the collaboration is excellent (FAC3)*,”

and highlighted how exposure to Ghanaian healthcare practice deepened their global understanding. Many noted that the experience reshaped their own pedagogical approaches, particularly emphasizing storytelling, contextual learning, and the spiritual and emotional dimensions of care.

Ghanaian faculty described significant learning from working alongside international colleagues, noting how these interactions broadened their perspectives on palliative care and strengthened their clinical and teaching skills:

“*Things I am not really considering because of the context… learning from how they practice will enhance my work (FAC2).”*

Across teams, faculty described moments of deep satisfaction, when learners connected concepts to practice, when reflective dialogue was exchanged, or when they themselves felt inspired by students' insights. One Canadian faculty reflected,

“*You kind of realise… I'm making a difference, not just with my patients, but with other patients I will never meet (PAC10).”*

Faculty also described overcoming initial insecurities, including fear of judgment or of being evaluated by peers. This vulnerability was ultimately reframed as part of an empowering and shared learning journey, where humility, curiosity, and openness catalyzed growth.

### Expanding the concept and future potential of co-creation

Participants consistently described co-creation as a model with significant potential beyond this single training program. For many, co-creation was a novel approach that, despite initial uncertainty, quickly demonstrated its value. The experience inspired faculty to consider broader applications, including additional courses, new clinical tools, research, guidelines, and learning materials. As one faculty put it,

“*We can think of developing more of such facilities… guidelines, tools, learning materials… for other learning purposes (FAC1).”*

The model also encouraged participants to envision new forms of globalized learning, where ongoing educational exchange extends beyond national borders. One faculty described this vision succinctly:

“*It is a more globalized form of learning… benefiting from different faculty even outside your own setting (FAC1).”*

### Barriers to co-creation and course implementation

Despite the overall success of the co-creation process, faculty identified several barriers that shaped their experiences and influenced the implementation of the palliative care course. These barriers arose at interpersonal, structural, and logistical levels and often intersected with broader cultural and system level constraints.

A key interpersonal barrier was the initial fear and vulnerability associated with co-creation, described by both Ghanaian and Canadian faculty. Participants from both groups expressed discomfort with peer observation, uncertainty about the adequacy of their knowledge, and hesitation in sharing their work within a new collaborative context. While these experiences were shared across participants, the study was not designed to formally compare perspectives between Ghanaian and Canadian faculty; however, the presence of vulnerability across both groups suggests that this was not limited to one side of the partnership. One faculty described how

“...*it's fear… people are not comfortable when people are supervising their work (FAC2)*,”

highlighting how apprehension about evaluation could impede early collaboration. This sense of vulnerability sometimes stemmed from professional hierarchies or differing levels of experience across the Ghanaian and Canadian teams. Several Canadians were concerned they weren't prepared,

“*much of what to expect was unfamiliar for us, and…. maybe they feel we were being too anxious.” (FAC10)*

Faculty noted that successful collaboration required

“...*throw[ing] your ego out the door… be ready to learn and also be ready to share (FAC2)*,”

underscoring how emotional safety and openness were foundational to the co-creation process. Over time, psychological safety appeared to develop through repeated interaction, collaborative problem-solving, and reciprocal teaching structures. Faculty described how shared responsibility for content delivery, openness to feedback, and the normalization of uncertainty contributed to a more supportive and less hierarchical learning environment.

Structural constraints also posed significant challenges, particularly limited time for pre course coordination. Faculty frequently reported that they wished for earlier interaction with their teaching partners, noting that

“...*it could have been more helpful to have… more one-on-one communication with the various faculty to align our planning (FAC10)*.”

Insufficient time for joint preparation hindered opportunities to harmonize teaching approaches, review culturally sensitive content, and fully integrate contextual adaptations in advance. These tensions were compounded by competing heavy clinical workloads and short timelines for preparing the modules.

Logistical barriers were another prominent theme, especially related to technology and connectivity. Multiple faculty described difficulties with internet reliability during virtual planning sessions and real-time classroom activities. As one faculty noted,

“...*most of the communication was Zoom, and sometimes we didn't have a very good network to interact (FAC2)*,”

while others emphasized that,

“...*the challenge here… is the internet (FAC6)*”

These disruptions affected the flow of both team planning and interactive learning, requiring rapid troubleshooting and flexibility from faculty and staff.

**Figure 1:**
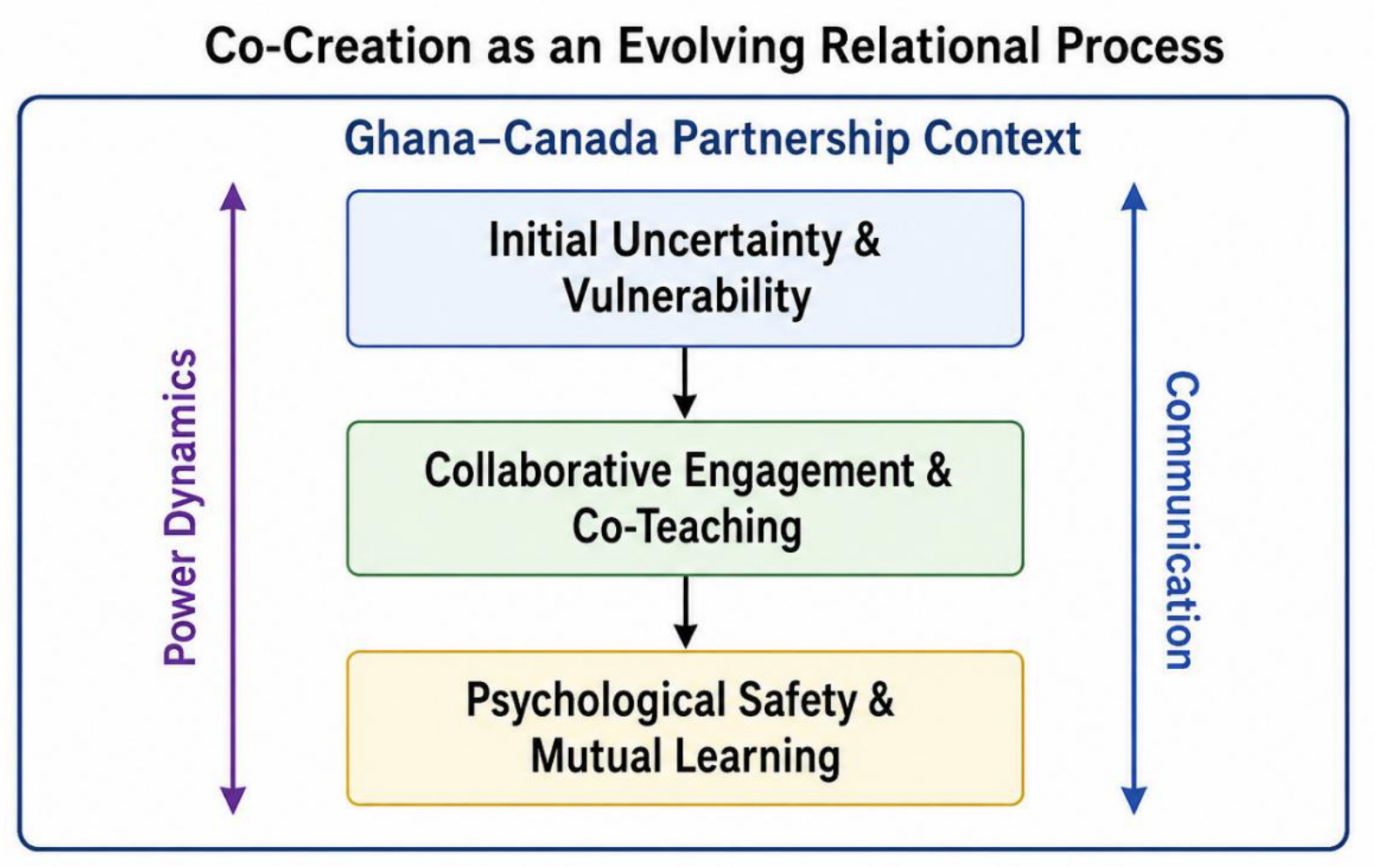
Conceptual model of co-creation as an evolving relational process. This figure illustrates the progression from initial uncertainty and vulnerability toward collaborative engagement and the development of psychological safety and mutual learning. These processes occur within the broader Ghana–Canada partnership context and are shaped by cross-cutting influences, including power dynamics and communication.

A conceptual model illustrating the relationships between themes is presented in Figure 1.

## Discussion

The aim of this study was to explore the lived experiences of Ghanian and Canadian faculty jointly developing and implementing or “co-creating” a palliative care course. This qualitative study demonstrates that co-creating a palliative care education program between Ghanaian and Canadian faculty is a deeply relational, iterative, and context dependent process that extends far beyond curriculum design. The five themes identified reinforce, extend, and in some cases complicate existing literature on global palliative care education, participatory curriculum development, and equitable international partnerships. Each theme reflects a distinct but interconnected dimension of co-creation, including contextual adaptation, relational development, communication, and professional growth. These findings should be interpreted within the context of this specific partnership and may not be generalizable to other settings.

### Contextual adaptation as the core mechanism of effective co-creation

Consistent with prior research in Ghana, Sub-Saharan Africa, and Indigenous and Latin American contexts (4, 6, 9, 18, 19), our study found that contextual adaptation is not a peripheral consideration but central to curriculum relevance and uptake. However, unlike previous studies that primarily focus on curriculum outcomes or implementation, our findings highlight the lived, real-time processes through which adaptation occurs during teaching.

This theme encompassed several related processes, including real-time clinical adaptation, negotiation of cultural relevance, and the use of contextually grounded teaching strategies such as storytelling. Faculty frequently described the need to revise clinical examples, reconsider drug availability, and recalibrate cultural assumptions in real time. While prior literature generally frames contextualization as a design principle, our findings illustrate the lived pedagogical labor of contextual adaptation with faculty immersed in moments of uncertainty, pausing, and recalibration during active teaching. This underscores that co-creation is not merely a value-driven commitment rather it is the practical mechanism through which curricula become clinically appropriate and culturally resonant.

Our findings align with prior studies emphasizing the importance of contextual adaptation in global palliative care education, including work in Nigeria and other LMIC settings, which highlight the need to tailor curricula to local clinical, cultural, and health system contexts (9). However, while much of the existing literature focuses on curriculum outcomes or implementation, our study extends this work by providing a detailed examination of the relational and interpersonal processes underpinning co-creation.

### Co-creation as a relational, iterative, and emotionally complex process

An important finding relates to the role of vulnerability and psychological safety in enabling co-creation (17). Faculty from both Ghanaian and Canadian contexts described initial uncertainty, fear of judgment, and discomfort with peer observation. While such vulnerability could reflect underlying power asymmetries inherent in transnational partnerships, the fact that it was expressed across both groups suggests that co-creation may disrupt traditional hierarchies by positioning all participants as both learners and contributors. Psychological safety appeared to emerge not as a precondition but as an outcome of the process, facilitated by repeated interaction, shared pedagogical responsibility, reciprocal knowledge exchange, and openness to uncertainty (20). These findings suggest that deliberately structuring opportunities for early engagement, mutual feedback, and co-teaching may be critical for fostering safe and effective collaborative learning environments in global health education. At the same time, we acknowledge that power dynamics related to institutional, cultural, and resource differences may still have shaped these experiences in ways that were not fully captured in the data. Future research should more explicitly examine how power is negotiated within co-creation processes, particularly in North–South partnerships.

The study strongly aligns with participatory action and co-design literature emphasizing iterative cycles of feedback, refinement, and shared decision-making (18, 21). International partnership driven scholarship also highlights the necessity of long-term relational engagement rather than unidirectional “import–export” models (22, 23). Our findings deepen this evidence base by elucidating the emotional and interpersonal dimensions of co-creation, including early uncertainty, fear of critique, and professional vulnerability, with the gradual movement toward trust, resilience and shared identity. Faculty's descriptions of “becoming more like a family” or needing to “throw your ego out the door” illuminate experiences that are rarely described in the literature but that significantly shape the quality and impact of co-creation. These insights suggest that protected pre-course time for relationship building is not optional but foundational for effective collaboration to develop sophisticated intercultural competencies that go beyond clinical expertise.

### Communication as the bridge between pedagogy, practice, and partnership

The findings support extensive evidence that communication is a central target of palliative care education and a critical driver of learner confidence and clinical practice (9, 24, 25). While prior studies emphasize the importance of communication in clinical training, our findings extend this by demonstrating its central role in enabling collaboration and trust within transnational teaching teams. At the same time, this study expands the conversation by demonstrating that communication is also the infrastructure of the co-creation partnership itself. Faculty relied on open, frequent, and reflexive communication to navigate cultural differences, negotiate teaching responsibilities, troubleshoot challenges, and build mutual trust. This dual role, communication both as a skill in content and collaborative processes, highlights its ethical and relational significance in transnational palliative care education and suggests that communication training should extend beyond learners to encompass faculty partnership development.

### Mutual professional and personal growth as a product of co-creation

In alignment with participatory palliative care studies showing bidirectional learning and strengthened capacity (18, 19, 21, 24, 25), participants found that co-creation facilitated substantial professional and personal growth for both Ghanaian and Canadian partners. Faculty reported expanded clinical perspectives, renewed pedagogical approaches, and deeper appreciation for the cultural, spiritual, and emotional dimensions of care. Notably, our findings contribute new insights by emphasizing how co-creation reshapes faculty identity and vulnerability and fosters professional confidence and resilience. These forms of transformation and growth are less frequently documented in the literature but underscore how co-creation benefits not only learners and institutions but also the educators themselves.

### Technology's role and limits in supporting co-creation

While literature from Africa and other low-resource settings highlights the promise of technology-enabled learning models (3, 9), this study illustrates the everyday friction required to make them function. Internet instability created barriers to both planning and delivery. These findings align with studies demonstrating that the success of virtual models depends heavily on connectivity and coordination (3). Our results therefore caution against overly optimistic narratives about virtual partnerships and highlight the need for robust technological and institutional support structures.

### Co-creation as an equity-oriented partnership practice

Global health education literature increasingly calls for partnerships that actively resist residual colonial dynamics by emphasizing reciprocity, local ownership, shared benefit, and reflexivity (22, 23, 26–28). The co-creation model described here aligns with these principles in practice: faculty emphasized shared responsibility, mutual learning, and “adding voices” rather than importing expertise. Although explicit decolonial terminology did not dominate participant narratives, the lived experiences of collaboration reflect many of the relational and power sensitive dynamics promoted in equity frameworks. The findings suggest opportunities for future work to make equity considerations more explicit and to continue examining the day-to-day practices that sustain equitable partnerships.

### Addressing evidence gaps through a focus on process

This study contributes to several recognized gaps in the literature. Few studies have examined transnational co-creation in the context of palliative care education in Ghana, and even fewer have foregrounded faculty experiences of primary care and rural contexts (2, 3, 6, 8). Our findings offer a detailed account of how co-creation unfolds across cultural, clinical, and institutional boundaries. While consistent with broader limitations in the field, such as the inability to link faculty experience to downstream patient or system outcomes (9), this work's primary contribution lies in elucidating the *process* of co-creation itself. These insights can inform future implementation research and support the development of more contextually grounded and equitable palliative care capacity building initiatives. This extends existing literature by providing a detailed account of how these processes are experienced by faculty engaged in co-creation within a transnational partnership.

### Limitations

This study has several limitations that warrant consideration. First, the findings are based on a small sample of 10 faculty participants from a single transnational partnership between two institutions. Although this reflects the pool of eligible faculty involved in the co-creation initiative, the limited sample size may constrain the breadth of perspectives captured and the transferability of results to other contexts or disciplines. While the diverse professional backgrounds of participants enriched the data, additional perspectives, particularly from larger or multi-site partnerships, may provide further insight into variability in experiences. Second, the study relied on self-reported experiences, which may be influenced by social desirability, interpersonal dynamics within the partnership, or the novelty of the co-creation model. Similarly, the participating faculty were a select group who had already demonstrated a pre-study willingness to participate in the co-creation model and thus may have been more likely to react positively to the process. Third, while the analysis offers rich insight into the processes and interpersonal dynamics of co-creation, it does not evaluate downstream outcomes for learners, clinical practice, or patient care, gaps that mirror broader limitations in global palliative care education research. Fourth, the program was well supported by a robust financial granting agency which facilitated both virtual and in person engagement of faculty which could constrain the replication of similar co-creation endeavors. Technological and logistical constraints, including intermittent internet access and limited time for pre course collaboration amidst competing academic and clinical priorities, may have affected both the experience of co-creation. The study was not designed to examine differences in experience between Ghanaian and Canadian faculty; therefore, we were unable to fully explore how power dynamics may have differentially shaped perceptions of vulnerability and psychological safety. Finally, although reflexive strategies were employed, the involvement of some researchers as faculty participants may have influenced interpretation despite efforts to mitigate potential bias. As the study included almost the full cohort of faculty involved in the program, the sampling strategy was comprehensive within this context but limited in scope, which may affect the transferability of findings to other settings. These limitations suggest the need for future research employing longitudinal designs, triangulation with learner and patient level outcomes, and comparative studies across diverse global partnerships.

These findings have several implications for practice, education, and research. For practice, they highlight the importance of intentionally fostering relational processes, such as trust, open communication, and psychological safety, within co-creative partnerships. For educational initiatives, the findings suggest that co-creation approaches may benefit from structured opportunities for dialogue, reflection, and shared teaching experiences, particularly in transnational collaborations.

The findings may also be transferable to other global health education partnerships that involve collaboration across differing cultural, institutional, and resource contexts. While the specific dynamics observed in this study are shaped by the Ghana–Canada partnership, the underlying processes of co-creation, including the negotiation of power, development of trust, and importance of relational engagement, may be relevant in other co-creative or collaborative educational settings. However, these insights should be adapted to local contexts and not assumed to be directly generalizable.

Future research is needed to explore how these relational dynamics unfold in other partnership models and settings, including larger or multi-site collaborations, and to examine how co-creation processes evolve over longer periods of time.

## Conclusion

This study offers insight into the experiences of faculty engaged in the co-creation of a palliative care education program within a Ghana–Canada partnership. The findings highlight the central role of relational processes, including vulnerability, communication, and the development of psychological safety, in shaping collaborative educational work. These dynamics were not static but evolved over time, underscoring the importance of sustained engagement and mutual learning within co-creative initiatives.

Importantly, these findings are grounded in the specific context of this transnational partnership and reflect the perspectives of faculty directly involved in the program. While the study provides insight into co-creation as a collaborative approach to global health education, the processes identified should be interpreted in relation to the cultural, institutional, and interpersonal contexts in which they occur.

Taken together, the findings contribute to a more nuanced understanding of co-creation by foregrounding its relational and experiential dimensions, offering considerations for future educational partnerships seeking to engage in collaborative, context-responsive approaches.

## Data Availability

The datasets presented in this article are not readily available because Transcripts of interviews may provide identifiable content. Requests to access the datasets should be directed to kirsten.wentlandt@uhn.ca.
